# Induced Pluripotent Stem Cells in Psychiatry: An Overview and Critical Perspective

**DOI:** 10.1016/j.biopsych.2021.04.008

**Published:** 2021-09-15

**Authors:** Alejandro De Los Angeles, Michael B. Fernando, Nicola A.L. Hall, Kristen J. Brennand, Paul J. Harrison, Brady J. Maher, Daniel R. Weinberger, Elizabeth M. Tunbridge

**Affiliations:** a Department of Psychiatry, University of Oxford, Oxford, United Kingdom; b Oxford Health NHS Foundation Trust, Oxford, United Kingdom; c Graduate School of Biomedical Science, Icahn School of Medicine at Mount Sinai, New York, New York; d Nash Family Department of Neuroscience, Icahn School of Medicine at Mount Sinai, New York, New York; e Department of Genetics and Genomic Sciences, Icahn School of Medicine at Mount Sinai, New York, New York; f Department of Psychiatry, Icahn School of Medicine at Mount Sinai, New York, New York; g Lieber Institute for Brain Development, Baltimore, Maryland; h Department of Psychiatry, Johns Hopkins University School of Medicine, Baltimore, Maryland; i Solomon H. Snyder Department of Neuroscience, Johns Hopkins University School of Medicine, Baltimore, Maryland; j Department of Neurology, Johns Hopkins University School of Medicine, Baltimore, Maryland; k Department of Genetic Medicine, Johns Hopkins University School of Medicine, Baltimore, Maryland

**Keywords:** Bipolar disorder, Drug screening, Model system, Neuron, Pathophysiology, Schizophrenia

## Abstract

A key challenge in psychiatry research is the development of high-fidelity model systems that can be experimentally manipulated to explore and test pathophysiological mechanisms of illness. In this respect, the emerging capacity to derive neural cells and circuits from human induced pluripotent stem cells (iPSCs) has generated significant excitement. This review aims to provide a critical appraisal of the potential for iPSCs in illuminating pathophysiological mechanisms in the context of other available technical approaches. We discuss the selection of iPSC phenotypes relevant to psychiatry, the information that researchers can draw on to help guide these decisions, and how researchers choose between the use of 2-dimensional cultures and the use of more complex 3-dimensional model systems. We discuss the strengths and limitations of current models and the challenges and opportunities that they present. Finally, we discuss the potential of iPSC-based model systems for clarifying the mechanisms underlying genetic risk for psychiatry and the steps that will be needed to ensure that robust and reliable conclusions can be drawn. We argue that while iPSC-based models are ideally placed to study fundamental processes occurring within and between neural cells, they are often less well suited for case-control studies, given issues relating to statistical power and the challenges in identifying which cellular phenotypes are meaningful at the level of the whole individual. Our aim is to highlight the importance of considering the hypotheses of a given study to guide decisions about which, if any, iPSC-based system is most appropriate to address it.

Psychiatric disorders are challenging to study mechanistically, as they are human specific and result from the complex dysfunction of a largely inaccessible tissue. Accordingly, putative brain cells derived from human induced pluripotent stem cells (iPSCs) have been enthusiastically adopted as potential model systems, as they have genomes of real people and can be manipulated experimentally. Many recent reviews cover their possible advantages and applications, both clinically and as research tools (1,2). However, few have critically appraised their use and attempted to situate them in the wider technical and conceptual landscape of psychiatric research (Figure 1). We attempt to do so here and provide some guidance for the design and reporting of iPSC studies in psychiatry (Table 1).

**Figure 1:**
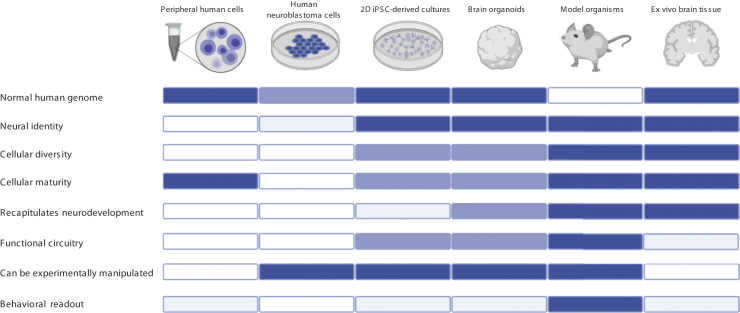
The strengths and limitations of different models of the intact human brain. The strengths and limitations of six different model systems—human cells obtained peripherally from living donors (or immortalized versions of these cells, e.g., lymphoblast lines), neuroblastoma cells grown in standard cell culture, 2-dimensional (2D) and 3-dimensional induced pluripotent stem cell (iPSC) models (brain organoids), animal models, and ex vivo human brain tissue (obtained either postmortem or resected during surgery)—are presented. Each is rated for each of the factors listed left, with the intensity of the color bar indicating the relative fidelity of each model. For example, cells and tissues derived from humans (including iPSCs) carry the genome of the donor, while the genome of human neuroblastoma lines contains various structural rearrangements, and animal models carry their species’ genome (107). In contrast, animal models are the only system in which behavior can be dynamically linked to invasive measures of cellular and circuit function, although it is possible to correlate phenotypes observed in human cells and tissues with behavioral phenotypes observed in the donors from whom they were derived. The other factors are discussed in more detail in the main body of the review. Note that the color bars are intended to give a broad, rather than a definitive, indication of model fidelity: there is likely to be significant variation within categories, particularly in the case of iPSC models, given the diversity of methodological approaches [e.g., co-culturing iPSC-derived neural progenitors with astrocytes accelerates maturation (31)]. Created with BioRender.com.

**Table 1:** Recommendations for iPSC Studies in Psychiatry

In Experimental Design	In Publication
Consider whether an iPSC-based model is the optimal system for the specific research question (see Figure 1). Consider the following: • Are neural cells required? • Are human cells needed? • Are living cells/tissue required? • Can the question be answered in immature cells/tissue? • Does the question require access to intact, complex neural circuitry?	Ensure that iPSC lines and methods are clearly and fully described, including: • The iPSC source (to allow readers to ascertain overlap between studies). • The number of donors included (and distinct donor lines, where relevant). • The number of technical replicates and measures of their variability, including both the number of differentiations, and the number of wells/organoids), as appropriate. • The differentiation protocol.
Determine the most appropriate iPSC system for the experiment in question, bearing in mind: • Selection of the type of cell, method of generation, considerations of purity, timepoint, inter- and intraindividual and interexperiment variation in vitro. • The extent to which the clinical trait is heritable and lifetime stable, taking into account whether the causal cell types underlying disease risk and progression are known. • Whether or not the phenotype is cell autonomous (i.e., observed at the level of an individual cell, without requirement for interactions with other cells). • The technical approach required to assess the phenotype (see Figure 2). • The level of cellular complexity and maturity required.	Assess and report the cellular mix comprising the iPSC model system, and how this varies between technical replicates and donors.
Identify the most meaningful phenotype (or range of phenotypes), mindful that one size is unlikely to fit all.	Perform and report power calculations for between-subjects analyses.
Ensure that between-subjects studies are appropriately powered. We recommend for such studies that researchers prioritize adding additional donors over multiple lines from the same donors to maximize statistical power (52).	Where possible, in the case of gene expression studies, attempt to validate iPSC-based findings using publicly available gene expression data from human postmortem brain, particularly that obtained from fetal tissue.
Where isogenic controls are to be used, assess the genetic manipulation of interest in lines from multiple donors, to assess consistency across genetic backgrounds (52).	Conclusions should be reported with appropriate caution and moderation, mindful of the limitations of current iPSC systems, and the challenges associated with validating their findings in vivo.
Design and conduct iPSC studies in a manner designed to maximize reproducibility (see Box 1).	

These suggestions are not intended to be proscriptive, but rather are intended to summarize and highlight the key issues that merit discussion and consideration, and our approach to them.

iPSC, induced pluripotent stem cell.

As we discuss below, iPSC systems are extremely well suited to experiments investigating fundamental molecular and cellular processes and, potentially, aspects of neurodevelopment. However, their utility in other situations is substantially less clear-cut. This is particularly the case for studies that seek to link cellular measures with psychiatry-relevant phenotypes observed in the whole individual, which, by their nature, are typically complex and temporally dynamic. Some of these challenges are purely logistical—relating to sample size and statistical power, for example—but others are more conceptual; for instance: even if robust relationships are observed between phenotypes at the cellular and whole-individual levels, how do we determine which (if any) are meaningful? We recommend that both nascent and established iPSC researchers return to the hypothesis that they seek to test and ask themselves these fundamental questions: what is it you want to model, and is the proposed iPSC platform able to achieve this with high fidelity? We believe that reflecting on these questions is critical for determining whether iPSC-based systems can help disentangle that which is meaningful from that which is merely possible.

## iPSCs in Psychiatry: A (Very) Brief History

The scale and complexity of iPSC studies has changed as the field has matured. Initial studies focused on monogenic conditions (3), while later studies compared iPSC neurons derived from patients versus control subjects (4). Researchers have also used patient-derived iPSC neurons to explore the potential cellular correlates of medication response (5). However, all these studies were conducted in extremely small samples (group sizes of 3–6) using a between-subjects approach, raising concerns about statistical robustness and generalizability. Therefore, more recent studies have moved toward investigations using isogenic lines to control for interindividual differences in genetic background (6, 7, 8, 9). Nevertheless, variable methodological approaches are used across the field and, in most cases, there is little or no evidence for replication of observed findings between cohorts and/or laboratories (see Box 1).

Box 1 **How Can We Maximize the Reproducibility of iPSC Models?**

Even prior to the introduction of experimental manipulations, there is inherent variability in induced pluripotent stem cell (iPSC) models—encompassing effects of genetic background, somatic mutations, and nongenetic factors—that can accumulate across derivation and differentiation processes if not carefully controlled. Furthermore, these different factors that can interact in complex ways. Large-scale studies have examined the sources contributing to variability in 2-dimensional iPSC cultures [reviewed in (108,109)]. Genetic background is the greatest source of variability between iPSC lines, although biological (e.g., age of donor) and technical factors (e.g., batch effects) also contribute (54,60,110). However, there is also marked heterogeneity between lines obtained from the same donor, and this within-individual heterogeneity varies between donors and therefore presumably results, at least in part, from genetic differences (60,110). Intriguingly, the subset of genes showing the highest variability both within and between individuals is enriched for targets of the polycomb repressor complex and H3K27me3, which in combination mediate epigenetic regulation of developmental processes, suggesting this pathway as a key mediator of both within- and between-subjects variability (110). A further source of variability is the potential presence of somatic mutations; many of these likely occur as the result of ultraviolet damage to donor fibroblasts, but they can also arise during derivation, giving rise to subclonal populations (111). Finally, even with standard operating procedures in place, nongenetic factors can result in substantial variation between laboratories (112). Crucially, all these sources of variability might plausibly be magnified later in derivation/differentiation and/or may manifest in a cell fate–dependent manner (108).

There are many steps that iPSC researchers can take to minimize the impact of the confounds outlined above on their research questions [see (108) for detailed recommendations]. Depending on the research question, the use of gold standard lines (including as the background for isogenic lines) may be desirable. Alternatively, where between-group differences are the primary comparison of interest, it may be appropriate to match groups for demographic factors (e.g., age, sex, ancestry). Irrespective of cellular background, quality control processes (e.g., karyotyping, single-cell RNA sequencing approaches) and the use of standardized protocols and meticulous record keeping remain critical: ultimately, the goal is to fully understand the lines used for a given experiment, and how this information might impact on a specific experiment.

A more recent development is the establishment of 3-dimensional (3D) cultures, termed brain organoids or spheroids. A few studies have compared 3D models derived from patients [with autism (10), schizophrenia (11), or bipolar disorder (12)] versus control subjects. Researchers are beginning to use organoids to explore the impact of genes harboring rare, penetrant disorder-associated mutations on neurogenesis and gene expression (13,14). Given their appeal as a potential window into human neurodevelopment and the early stages of psychiatric disorder pathology, it is likely that studies in organoids will become more widespread. However, most psychiatric organoid experiments remain small in scale and so essentially represent proof-of-principle studies.

## Psychiatric Illnesses in a Dish? The Questions of Phenotype and Cell Type

The potential of iPSC models is to approximate biological aspects of illness or risk in the context of a particular human cell, with a particular human genome, which may then be studied to illuminate pathogenic mechanisms and to identify targets for remediation or rescue. Arguably the biggest challenge in realizing this potential is to identify the most appropriate phenotype(s) to measure and the most suitable cell types to study. How straightforward this is will depend on the hypothesis as well as on practical and technical constraints (note that even hypothesis-free/generating studies still require decisions, e.g., about donor/cell type). For studies of specific genes, the phenotypic readout may be clear, at least theoretically. For example, if the goal is to examine the effect of (rare) disease-associated coding mutations in an ion channel gene, it makes sense to determine the properties of the resulting channels (15) and the function of downstream effector pathways. Similarly, iPSC-based models are extremely well suited for the study of fundamental cellular processes, particularly those that require cells to have a neural identity, such as dendritic outgrowth and synapse formation. However, it is often less obvious what the appropriate assay should be, particularly when the molecular target of interest is of unknown function; this can be the case even for well-studied genes if they give rise to multiple, functionally distinct products or are involved in a variety of molecular pathways (16,17).

Decisions about phenotype(s) are particularly challenging for case-control studies, as the absence of any pathognomonic neuropathological changes in patients with psychiatric disorders (unlike neurodegenerative diseases) means that there is no clear—let alone diagnostically significant—disease phenotype to model at the cellular level. Conversely, we do not know whether a phenotype in a rarefied cell model of early neuronal development would be expected to mirror characteristics of illness in mature brains, even if the cell model phenotype reflects a basic pathogenic mechanism. Several studies have demonstrated changes in dendritic outgrowth and synaptic density in patient-derived iPSC neuronal cultures (4,18), mirroring, at least superficially, evidence for alterations in neuropil and synaptic density reported in adults with some psychiatric conditions, particularly schizophrenia (19, 20, 21). However, other findings are less clearly allied to neuropathological observations. For example, it is hard to square the substantial reductions in neuronal viability observed in cultures derived from iPSCs from patients with bipolar disorder (22) with the limited evidence for changes in neuronal number in the brains of patients (23). Similarly, findings of disorganized migration of neural progenitor cells in brain organoids derived from patients with schizophrenia (11) are inconsistent with the lack of evidence for neuronal disarray in postmortem brain tissue (21,24). Clearly, it is possible that some of the case-control differences observed in iPSC systems occur transiently in the disease state during development. However, they may represent inaccurate renderings of genuine group differences found in vivo. For example, differences in neurotransmitter function in the mature brain may manifest as changes in migration in vitro, as many neurotransmitters act as trophic factors during development (25), while the lack of microglia, and concomitant reduction in synaptic pruning (26), in many iPSC models may confound phenotypes relating to synaptic size and/or number. Finally, group differences may also represent false leads, as the result of insufficient sample size or related to the vagaries of the in vitro environment (27), for example.

A major obstacle to the accurate modeling of neuropsychiatric disorders in vitro is that iPSC-derived neural cells are typically relatively immature (28). Gene expression and electrophysiological studies indicate that cells within both 2D and 3D models remain broadly similar to early fetal neurons (29, 30, 31, 32) [although organoids containing cells mapping to early postnatal development have been recently reported (33)]. Notably, although often cited as a limitation, the fetal-like identity of iPSC-derived neural cells may have some advantages, at least in theory. If they truly recapitulate aspects of human neurodevelopment (see below), they may provide a window into processes that are otherwise largely inaccessible. Therefore, for researchers focused on psychiatric disorders with a neurodevelopmental origin, such as schizophrenia (34) and autism (35), iPSC-based models may provide a system in which to study cause and effect relationships.

## Can iPSC Models Predict Whole Individual Phenotypes?

One of the appealing aspects of iPSC-based models of psychiatric disorders is their potential to identify cellular phenotypes that predict information about an individual’s illness course or medication response, including the wider penumbra of clinically relevant (endo)phenotypes, e.g., cognitive function. Accordingly, there is much interest in using cellular phenotypes as a functional readout for high-throughput drug screening to identify new therapeutic approaches in a target-agnostic manner (“phenotypic drug discovery”) (36). For this potential to be realized, it will be important to identify phenotypes that are relevant to the illness experienced by those from whom the cells were taken and/or that reliably predict clinical improvement. The best-studied area is lithium responsiveness in patients with bipolar disorder, although examples exist for other psychiatric disorders, e.g., autism (37). Mertens *et al.* (5) demonstrated hyperactivity in iPSC-derived dentate gyrus neurons derived from patients, which was reversed by lithium only in those derived from lithium-responsive patients. In follow-up studies, the same group replicated these findings, successfully predicted the lithium responsiveness of a patient using a classifier trained on electrophysiological parameters of responder versus nonresponder cells (38), and suggested that hippocampal CA3 hyperexcitability might be unique to lithium responders (39) and reduced by lithium exposure (40). However, these studies used small samples and emerged from a single laboratory. As promising as this appears, it will be important to ensure that this phenotype is reliable in a larger sample and, crucially, robust across laboratories.

There has been a tendency in some reports to overinterpret findings in the context of loosely linked clinical phenomena. For example, alterations in catecholamine release have been reported in iPSC dopamine neurons derived from patients with schizophrenia, compared with control subjects (41), and have been taken as evidence for consistency between in vivo and in vitro measures (42), arguing that in schizophrenia there is excessive DA release in striatum. However, changes in dopamine synthesis fluctuate within individuals over time (43) and track the phase of the illness (44), suggesting that it is a marker that is (at least in part) “state,” rather than “trait.” Although there is extensive wiping of epigenetic (and therefore presumably “state”) marks during reprogramming to the iPSC state, it may be incomplete in some circumstances (45,46), including at least one locus of relevance to psychiatry (the fragile X locus, *FMR1*) (3). It will therefore be crucial to assess whether and how these residual signatures are related to environmental and “state” markers, including fluctuations in the donor phenotype. This understanding is critical if we are to understand how (and if) iPSC models, taken at a relatively few moments in the changing developmental landscape of early cell differentiation, can predict anything meaningful about the aspects of the illness in those from whom the cells were taken.

In conclusion, because there is no single cellular phenotype relevant to all situations in psychiatry—or even for any specific disorder—the decision as to what to study in iPSC-based systems is unclear. Indeed, we would argue that approaching studies from a standpoint of “what to study?” is fundamentally misguided in many cases. Arguably, the relevant question in most situations is not “what phenotype is best placed to identify group differences?,” but rather “what phenotypic readout is best suited to provide meaningful information about my manipulation/comparison of interest?” An examination of the literature suggests that group differences are relatively easy to detect at the cellular level—we are unaware of any negative study in psychiatry (although this may, of course, be due to publication bias)—but the importance of this observation remains opaque. It is possible that iPSC-based systems, freed from the selection pressures and compensatory mechanisms that operate at the level of larger-scale neural systems, are uniquely placed to identify relationships between cellular phenotypes and manipulations of interest. However, it remains a significant challenge to determine which of these relationships are meaningful in terms of pathophysiology or prognosis. Notably, it is also possible that some disease-relevant cellular processes are only observed in the context of physiological stimuli. Ultimately, the onus is on individual investigators to be fully transparent about their motivations and how this relates to their findings. Hypothesis-free studies can be extremely valuable for generating novel hypotheses for follow-up testing, for providing a resource for the community (47, 48, 49, 50, 51), and potentially, for identifying predictors of patient symptoms/outcome. Nevertheless, post hoc explanations retrofitted to cellular observations are of much less value and in the long run have the potential to damage the reputation of the very model systems they seek to advance. iPSC systems are best viewed as being another tool in the psychiatry researcher’s armory; they have potentially unique utility for understanding fundamental cellular processes and simple network activity but are less suited for investigations of intact brain systems and, crucially, how complex brain functions change across the dynamic neurodevelopmental landscape.

## How Many Lines Are Enough? Ensuring Robustness and Reproducibility

Traditional cell culture experiments typically examine the effect of an experimental manipulation in a standard cell line, meaning that only technical replicates—i.e., repeated measurements within the same sample—can be obtained. In contrast, iPSC experiments can investigate multiple sources of variation: technical (i.e., how consistent are the effects of individual experiments given the same starting iPSC-derived cells?), within-subjects (i.e., how consistent are results from different iPSC lines derived from the same donor?), and between-subjects (i.e., how consistent are findings across individuals?). The ability to study differences between groups, or the impact of a given manipulation on a range of different genetic backgrounds, has great potential for understanding the differences between individuals. However, it also raises important caveats regarding sample size and power (52).

**Figure 2:**
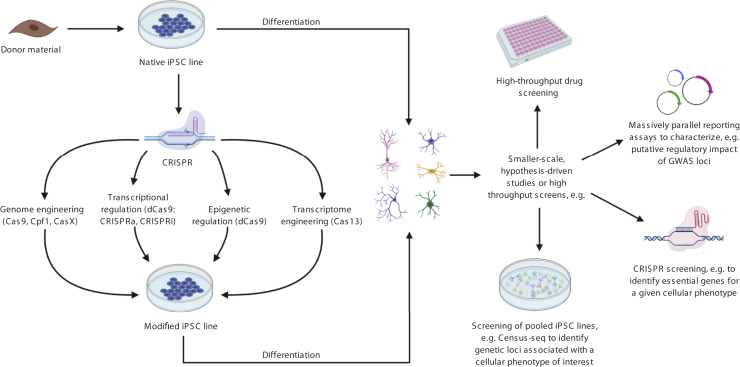
The diverse applications of human induced pluripotent stem cell (iPSC) models. Rapid developments in genome engineering (e.g., CRISPR [clustered regularly interspaced short palindromic repeats]) and iPSC-compatible systems means that human iPSC-derived models can be employed in a large number of ways in psychiatry. On the left of the figure is an overview of the many aspects of genomic and transcriptomic factors that can be modified using different forms of CRISPR [detailed in (2)]. Both native and edited cells can then be used for a wide variety of applications; examples are shown on the right. [See (113) for drug screening, (114) for massively parallel reporter assays, (115,116) for CRISPR screening; (55) for Census-seq]. To date, most studies have employed 2-dimensional iPSC systems; however, many of these approaches, e.g., CRISPR screening (117), are also compatible with 3-dimensional systems. Created with BioRender.com. GWAS, genome-wide association study.

In common with all between-subjects approaches, *n* for the purposes of iPSC-based studies remains the number of lines obtained from different individuals, rather than different iPSC lines taken from a single donor or technical replicates. Given this constraint, studies that pool lines from multiple donors are becoming increasingly prominent (Figure 2) and permit cost-effective resolution of expression quantitative trait loci (eQTLs) (53,54) and genome-wide association studies of cellular phenotypes (55). However, pooling is unsuitable for detecting donor-autonomous phenotypes and is currently only compatible with limited functional readouts (single-cell RNA sequencing [RNA-Seq], whole genome sequencing, or single nucleotide polymorphism [SNP] array) as individual donors within cellular villages must be disambiguated based on genomic information. While technical advances aim to improve the scalability of single-donor iPSC studies (56, 57, 58), working with multiple lines simultaneously remains a logistical challenge owing to the significant length of time needed per differentiation and the infrastructure (e.g., incubator space, personnel) required for larger-scale experiments. Therefore, large iPSC studies will likely involve staggered differentiations, which in turn necessitates the careful control of batch effects (59) and limits the number of distinct lines that can feasibly be included in a single experiment. Large-scale iPSC studies are emerging (53,60, 61, 62), but even the biggest single study conducted to date used lines from fewer than 350 subjects. This poses significant challenges for case-control studies in psychiatry (and, indeed, for all complex disorders) in which, at least at the level of the whole individual, biological effects of risk factors are small, meaning that large numbers of individuals, and careful phenotyping (e.g., high-depth RNA-Seq), are needed to detect them (52). It is plausible that group differences are more penetrant at the cellular level, as cellular phenotypes are “closer” to the substantial genetic risk that underpins psychiatric disorders, but this remains to be proven. Furthermore, significant questions remain about the reproducibility of findings (see Box 1), as few studies include attempts at replication. Notable diversity remains in differentiation protocols and other methodological considerations across the field (63, 64, 65, 66); in the absence of standardized protocols, it is unclear how to interpret differences observed between methodologically distinct studies.

In cases in which a within-subjects control can be used (e.g., an isogenic control, for genetic manipulations, or a vehicle control, for pharmacological interventions), it may be possible to draw conclusions using only a few (~5–6) individual iPSC lines to give acceptable confidence that findings are consistent across genetic backgrounds (52). In contrast, when the comparison of interest is between groups of subjects, it will typically be most appropriate to prioritize the study of iPSC lines obtained from different individuals over replicate lines from the same individual. Thus, the scale and complexity of an experiment will need to be guided by the key questions that it seeks to answer to deliver robust findings.

## Are 3D Models the Future?

Psychiatric symptoms are associated with subtle and widespread changes in brain function; thus, the ideal model system would permit the study of relatively complex circuitry and interactions between regions. Organoids are therefore appealing as a relatively complex human neural system that are compatible with many existing technical approaches and can be readily manipulated (12,67).

Many protocols aim to produce cortical tissue (63,68,69) including the hippocampus, but models of other brain regions exist, including the midbrain (68,70) and cerebellum (71). Furthermore, a choroid plexus organoid has been recently developed, raising the exciting prospect of a novel in vitro model for testing for brain penetrance during drug development (72). Organoid models are not as widely used as 2D iPSC culture systems and are technically challenging to establish and analyze. However, there are several noteworthy limitations and unanswered questions with regard to current systems.

Early protocols relying on self-organization gave rise to organoids that showed substantial variability in their cellular composition, tissue identity, and morphology (73). Technical refinements have improved reproducibility, although differences between batches and cell lines are still observed (73,74). It will likely be possible to refine protocols further; however, for the time being, heterogeneity remains a significant issue, and so it is crucial for experimenters to carefully design experiments to minimize its impact, for example, by averaging across multiple batches and using single-cell RNA-Seq approaches to understand the cellular milieu. Organoids cultured for extended periods can contain diverse cell types, including astrocytes, dopamine neurons, and multiple subtypes of forebrain neurons (73,75). However, they do not give rise to all of the major cell types found in human brain. For example, current organoids lack microglia and myelination (76), and many do not contain GABAergic (gamma-aminobutyric acidergic) interneurons (77), so it is unclear how faithfully their neural circuitry mimics that of the intact brain. iPSC-derived neural, microglial, and astrocytic precursors can integrate to form 3D cultures (78), allowing researchers to manipulate the cellular mix to fit their experimental needs. Nevertheless, the popular conceptualization of organoids as “mini-brains” remains far from accurate: their organization is limited and often unpredictable, and their complexity and cellular diversity is a fraction of that seen in vivo.

Given their potential utility for measuring the formation of neural circuits, a crucial outstanding question is the extent to which brain organoids faithfully recapitulate neurodevelopment. Current findings are mixed. They contain a complex mix of neural cells and exhibit a quasi-laminar structure similar to superficial and deep layers of the cortex, and exhibit network activity (75,79, 80, 81). The neurons within them can make long-range connections, both within the organoid and with neighboring structures in the culture system or in vivo in animals, and during long-term culture, they exhibit molecular switches that mimic those seen during neurodevelopment (33). However, a recent study suggested that brain organoid cells may show abnormal maturation, possibly as the result of cellular stress induced by the in vitro environment, and lack the fine spatial resolution seen in vivo (27). Thus, further studies are required to clarify which aspects of human neurodevelopment (if any) can be accurately modeled using organoids, emphasizing the need for caution when extrapolating from organoid findings to fundamental aspects of human neurobiology: it is certainly too early to conclude that findings from organoids provide proof for neurodevelopmental changes in vivo (11).

Researchers are using novel strategies to enrich for the cells types that are their focus [e.g., the recent development of oligodendrocyte spheroids (82)] and/or fusing different types of organoids into “assembloids.” Some fusions are designed to study interactions between neurons with distinct regional identities, combining organoids with dorsal and ventral forebrain identities to study interneuron migration (83), for example, or fusing cortical organoids with thalamic or striatal organoids to study the formation and function of long-range axonal connections (84,85). Brain organoids can also be fused with cell or tissue types that they lack (e.g., microglia or blood vessels) (86,87). Impressively, a recent study demonstrated that distinct organoids with cortical, spinal, and skeletal muscle identities can be successfully fused to form a functional corticomotor circuit (88). Assembloids have yet to be widely used in psychiatry, but examples are beginning to emerge, such as the demonstration of altered migration in assembloids derived from individuals with Timothy syndrome (89). Ultimately, bioengineering approaches may allow the spatially controlled delivery of external patterning cues, to further increase the sophistication and reproducibility of organoid and assembloid models (86,90). However, even the most advanced models will likely never rival the complexity of the human brain; even if this were ever technically feasible, it would raise substantial ethical questions (91).

## Can We Unravel Mechanisms of Genetic Risk Using iPSCs?

As the use of iPSC-based models in psychiatry has gained popularity, many researchers have shifted their focus from patient versus control studies to those seeking to understand the functional impact of disease-associated genetic risk. Rare and highly penetrant variants are particularly amenable to this approach, as their large effect sizes (92) mean that their impact can be studied between subjects in relatively small samples. Several groups have successfully used iPSC-based models to illuminate the impact of rare variants of relevance to psychiatry on neuronal function (7,8), underscoring the utility of iPSC-based models for understanding highly penetrant variants. However, this approach is also amenable to investigation of the summative effects of common variants on cellular function, as extremes of polygene risk scores approach the odds ratios of many rare variants (93).

Advances in genome engineering (2,94, 95, 96) allow researchers to study the impact of genetic variants of interest, or to manipulate specific transcripts or epigenetic factors, on an isogenic background, thereby removing variance owing to genetic differences between individuals [although the impact of variants should be studied on multiple lines to assess reproducibility and generalizability (52)] (Figure 2). However, even with the use of isogenic lines, studying individual common variants using iPSC models is more challenging than studying rare variants, as their effect sizes are far smaller (93). Moreover, it is typically unclear whether the SNPs identified by genome-wide association studies are directly relevant for pathophysiology, or whether they are simply in linkage disequilibrium with causal variants. Therefore, although appealing in theory, studies aiming to systematically (but agnostically) introduce genome-wide association study risk-associated alleles into iPSCs for functional screening are likely to be of limited use. Indeed, because most risk SNPs are noncoding, it is often not clear which the relevant gene or genes are, let alone how their function is altered in association with disease risk. We therefore suggest that researchers seeking to use iPSC models to investigate the biological impact of risk SNPs draw on information from the increasing number of large-scale studies investigating gene expression and function in native human cells and tissues, and the ways in which these factors are affected by genetic variation (47, 48, 49,51). Information of this type can be used not only to help to identify the specific genes tagged by individual risk SNPs (97), but also to clarify the pathophysiological mechanisms at a molecular level. It may then be more appropriate to model the molecular consequences of genetic variation, rather than to directly recapitulate the genetic variant itself: a strategy that may be particularly useful in cases in which the functional variant is unclear. For example, data obtained in postmortem human brain identified a small subset of schizophrenia risk loci that showed single-gene eQTLs; for one of these—rs4702 (93), in the furin gene—the schizophrenia-associated SNP is putatively the causal variant (98). Based on this information, Schrode *et al.* (6) modeled these single-gene eQTLs in iPSC-derived neurons. Strikingly, as well as individually influencing aspects of synapse development and neuronal activity, the combination of these distinct perturbations altered the expression of genes enriched for those differentially expressed in psychiatric disorders (6). These findings highlight the potential of using information from human samples to refine iPSC models in which to explore pathophysiological mechanisms.

iPSC systems might be anticipated to be better powered to detect associations between risk SNPs and aspects of gene expression than are postmortem studies, as a comparison of lines carrying risk SNPs versus nonrisk isogenic control SNPs should eliminate noise arising from genetic background and, potentially, from environmental and perimortem factors (52). However, even when the causal SNP is known, there are several other factors that may undercut the assumption that gene expression phenotypes will necessarily be cleaner in iPSCs than in human tissue. Some of these factors are technical in nature: for example, although iPSCs can be differentiated into a wide array of distinct cellular populations, they are not yet able to capture the full diversity of neural cell types, meaning that cell type–specific expression phenotypes (99) may be missed [although note that studies of bulk postmortem tissue can also fail to detect effects of SNPs on cell- and/or isoform-specific aspects of gene expression (100,101)]. Other factors result directly from the ex vivo nature of the iPSC approach: specifically, it is possible that environmental factors may be required for certain risk SNPs to mediate their pathogenic effects (102), and therefore that their effects may not be observed in the absence of these environmental factors. Last, eQTLs can vary across cellular differentiation (103) and neurodevelopment (104), and across brain regions and cell types (101), meaning that a causative risk–associated SNP may only have pathogenic implications in a particular cell population at a particular time of life. Thus, the relative merits of iPSC systems versus postmortem studies for identifying eQTLs remains to be determined. Given the diversity and complexity of relationships between individual SNPs and different facets of gene expression, it seems unlikely that either approach will be “superior” in all cases.

## Conclusions

iPSCs offer access to live human brain cells, provide the opportunity to study cause and effect relationships, and provide the means to investigate how these differ between individuals. Their compatibility with a wide range of technical approaches and their ready manipulation by genome engineering makes them, in many ways, the ultimate tissue culture system. They are particularly well suited to understanding how fundamental processes occurring in (and between) neural cells are affected by penetrant genetic risk factors, or by pharmacological and environmental manipulations. However, they are arguably less well suited to the study of group differences between psychiatry patients and control subjects, both because of the large numbers likely needed to achieve statistical power and because of the challenges of determining which cellular phenotypes are meaningful. Even if statistically robust phenotypes associated with psychiatric illnesses and/or genetic risk for them are identified, it will be a challenging endeavor to demonstrate their validity and relevance in vivo. This problem is particularly acute in the case of iPSC phenotypes that map onto early human neurodevelopment because there are currently no means to determine ground truth.

Many of the challenges currently faced by iPSC approaches are not unique: researchers working with postmortem brain tissue and with conventional cell lines have been grappling with similar questions for many years (105,106), and concerns regarding reproducibility are widespread. The impact of iPSC-based findings will undoubtedly be maximized by seeking convergence with those acquired using other approaches that provide insight into cellular and network phenomena, be they obtained using traditional cell culture, or research in human samples or animal models (Figure 1, Table 1). The field will be best served by researchers remaining focused on their hypothesis of interest and clear-eyed about the suitability of iPSC-based models for testing it.
